# Mastering life together - symptom management, views, and experiences of relatives of persons with CPTSD: a grounded theory study

**DOI:** 10.1186/s41687-018-0070-5

**Published:** 2018-10-19

**Authors:** Manuel P. Stadtmann, Andreas Maercker, Jochen Binder, Wilfried Schnepp

**Affiliations:** 1 Department of Health, University of Witten/Herdecke, Alfred-Herrhausen-Strasse 50, 58448 Witten, Germany; 2 Department of Psychology, Psychopathology and Clinical Intervention, University of Zurich, Binzmühlestrasse 14/17, 8050 Zurich, Switzerland; 3 Centre for Trauma Disorders, Integrierte Psychiatrie Winterthur, Technikumstrasse 81, Winterthur, Switzerland

**Keywords:** Complex posttraumatic stress disorder, CPTSD, ICD-11, Psychiatry, Grounded theory, Symptoms, Mental health, Relatives, Caregivers

## Abstract

**Background:**

Complex posttraumatic stress disorder is described as a chronic condition with several severe and concurrent symptoms. Symptoms influence and impair not only the affected individuals but also their social surroundings and their relatives. The literature describes relatives as a key factor in managing symptoms, both as a barrier and a facilitator.

**Aim:**

This research aimed to explore and to reconstruct the views, perceptions, experiences, facilitations and barriers of relatives who support the symptom management of persons with CPTSD in everyday life.

**Methods:**

A theoretical sampling was used to recruit for an interview 18- to 65-year-old relatives of patients with diagnosed CPTSD. The 17 semi-structured interviews were audio-recorded and transcribed verbatim. The transcriptions were uploaded into MAXQDA, and a Grounded Theory method based on that of Corbin and Strauss was used to analyse the data.

**Results:**

We provide a process model with 5 interacting phases: the initial situation, state of permanence, being an anchor, recognizing limits, and potential outcomes. Each phase is further divided into subcategories.

**Discussion:**

Participants experienced their condition as unpredictable. Although they mastered different strategies through own exploration and in cooperation, there is a clear need for more education, advice and support for relatives caring for those affected by CPTSD. Health care services should consider providing family support, educational services and increase the involvement of relatives in treatment. Over all, well-supported relatives can play a facilitative, key role in improving symptom management.

**Trial registration:**

Ethical approval was obtained from the Swiss Cantonal Ethic Commission (Nr 201,500,096). This research was also registered at the World Health Organization Clinical Trials Search Portal through the German Clinical Trial Register, Trial DRKS00012268.

## Background

The World Health Organization’s (WHO) 11th version of the International Classification of Disease (ICD-11) will supposedly be introduced in 2018 [1–4]. In line with the proposals of the WHO Working Group for Disorders Specifically Associated with Stress [5–7], the latest version will include the diagnosis of complex post-traumatic stress disorder (CPTSD). Several studies across multiple samples have provided evidence of a distinction between the diagnosis of post-traumatic stress disorder (PTSD) and CPTSD [1, 3–5, 8–16]. Criteria of PTSD diagnosis comprise a reaction to a traumatic experience and three symptom clusters: re-experiencing, avoidance, and a sense of threat [2, 4–7, 11, 16–18]. The core criteria for PTSD comprises first: re-experiencing the traumatic event in the present for instance in the form of flashbacks intrusions that were experienced during the traumatic event. Second: The presence of avoidance of thoughts, memories or places related to the event. Third and last the presence of persistent perceptions of heightened current threat, for instance hypervigilance. The symptoms must persist for at least several weeks and cause significant impairment in important areas of functioning [19]. Additional to the described symptom clusters, CPTSD diagnosis requires the presence of three further symptom clusters. Those three clusters described in the literature as symptoms of disturbances in self-organization (DSO) [1, 7, 9, 20–22]. The clusters are as follows: First, symptoms of affective dysregulation. For instance, difficulties in handling emotional outbursts or depressive symptoms. Second, symptoms concerning a negative self-concept, such as feelings of sever shame or severe guilt related to the traumatic event. Third, symptoms of problems in interpersonal relationships, for instance severe difficulties in sustaining relationships or problems in feeling close to others.

Recent literature provides first results concerning adverse factors of patients with CPSTD, such as poorer functioning [1, 16], unemployment despite higher education [9], living alone (Stadtmann MP, Maercker A, Binder J, Schnepp W: ICD-11 complex posttraumatic stress disorder, characteristics and symptoms of adults in an inpatient psychiatric setting: A descriptive study, submitted), financial difficulties (Stadtmann MP, Maercker A, Binder J, Schnepp W: ICD-11 complex posttraumatic stress disorder, characteristics and symptoms of adults in an inpatient psychiatric setting: A descriptive study, submitted), higher rates of comorbidity ([15]; Stadtmann MP, Maercker A, Binder J, Schnepp W: ICD-11 complex posttraumatic stress disorder, characteristics and symptoms of adults in an inpatient psychiatric setting: A descriptive study, submitted) and lower rates of secure social attachments [15]. Latest evidence provides evidence for further correlates such as anxiety and aggression [8] but also depressive conditions, negative trauma-related cognitions, the presences of reduced distress tolerance [21], and having experience childhood abuse in sexual, physical or other form [9]. A recent study provided evidence of the difficulties and facilitators in symptom management of patients with CPTSD in the context of social support (Stadtmann MP, Maercker A, Binder J, Schnepp W: Why do I have to suffer? Symptom management, views and experiences of persons with a CPTSD: A grounded theory approach, submitted). For the persons affected, social support from a relative was a key intervening factor (Stadtmann MP, Maercker A, Binder J, Schnepp W: Why do I have to suffer? Symptom management, views and experiences of persons with a CPTSD: A grounded theory approach, submitted).

According to the literature, there are different forms of social support [23–26], for instance, formal social support from professionals (e.g., nurses, psychologists, psychiatrists, and social workers) as well as informal social support from e.g., family members, friends or neighbours [24, 27, 28]. Individuals who provide informal support are usually relatives and are typically unpaid. The support encompasses assistance that is exchanged between individuals and is manifested in various forms, such as instrumental, informational and emotional assistance [24, 27, 28]. Instrumental support means help with financial issues, transportation, in the household and with administrative tasks. It is also the provision of advice, suggestions, and information. Emotional support refers to actions that make the individual feel loved and cared for, that promote trust towards another person and affirm the individual’s sense of self-worth.

In general, as informal social support, patients’ relatives play a significant and vital role in research on symptom management [29–33]. Relatives of those affected by a chronic mental illness such as CPTSD support them in several ways. For instance, in offering hope, providing encouragement, or developing opportunities [34]. They also provide support, for example, monitoring medications or offering help with administrative tasks or with domestic work [34]. Moreover, support can enable sufferers to learn new skills and strengthen their social relationships with others [35]. Additionally, several facilitators for social support have been described including motivation for caregiving, sense of purpose and validation and emotional experiences with those affected [36].

Again, there is a growing body of literature on caregiver burden and several studies highlight the difficulties and specific needs of relatives providing care and support for those affected by chronic mental illness [37–43]. For instance, the relatives’ struggle with the lack of supportive resources and the ensuing emotional exhaustion [37]. Caregiving tasks interfere with many other responsibilities [38], for example providing emotional support to the person affected, in addition to regular family duties [39], demands on time [36], being a reference for family matters, thus leading to feelings of loss of self [37]. Relatives may experience psychological distress due to a lack of supportive resources, for instance during times of financial stress [40]. Likewise there is a need for education and information as relatives generally have little prior knowledge of mental illness [42]. The process of caregiving and supporting may result in feelings of isolation, anxiety, anger and frustration [41]. Overall, providing social support can lead relatives of a person suffering from mental illness to experience a poor quality of life, which may in turn worsen the symptoms and condition of the person affected [43]. These results are in line with literature describing relatives as being both a facilitator and a barrier to patients’ symptom management [34, 44, 45].

The literature for PTSD patients and their relatives shows similar findings. Moreover, patients with PTSD would like their relatives to be more involved in their mental health care [46–48]. Social support thus appears to play an important and beneficial role in coping with symptoms of traumatic stress [49–52]. As stated by Maercker and Horn [33] in their “The socio-interpersonal model”, social support is proposed as a relevant factor for development or maintenance of PTSD and its recovery. Despite the positive effects this support can have on patients with PTSD, various difficulties for relatives of these patients were concurrently indicated. There appears to be an emotional impact of feeling lonely, unhelpful and stressed during the trajectory of the disease [53, 54]. Further social implications are also described, for instance feeling isolated and helpless with regard to the unpredictable nature of the condition [53]. Also dealing with emotional numbing and angry outbursts negatively impacts relationships [55, 56]. Providing care and support for relatives with PTSD can create a financial burden and deep psychological stress [54, 57]. Findings also show that wives’ sense of caregiver burden was associated with, first, the severity of their husband’s PTSD symptoms [58] and, second, the degree of impairment in his daily life as well as his occupational functioning [59]. No studies were found in the context of CPTSD research.

Overall, research on relatives’ support and symptom management is still developing [60–62]. However, addressing relatives’ needs may lead to their subjective stress being reduced and their levels of self-care and emotional role functioning increasing [63]. These effects could, in turn, lead to better support in symptom management and improve the condition of CPTSD patients. However, there is little evidence to this effect and to the best of our knowledge no corresponding study has investigated the role of relatives during symptom management of patients with CPTSD. This is the third study of a larger mixed method research project to investigate symptom management and the social process of persons affected with CPTSD and their relatives [64]. In our first quantitative descriptive study we were able to identify adult CPTSD inpatients. Further we provided results concerning their symptom burden and related characteristics. Such high prevalence of unemployment, high prevalence of childhood abuse, high prevalence of depressive symptoms and different levels of CPTSD symptom burden. Based on that results and we were able to identify inpatients for interviews, aiming to explore their symptom management in everyday life. At the same time a related relative was additional asked for an interview, aiming to explore their view, experience and their role in symptom management of those CPTSD affected. Therefore, the aim of this study is as follows.

## Aims

The goal of this study was to explore and reconstruct the everyday life views, perceptions, experiences, facilitations and barriers of relatives during the symptom management of those with CPTSD.

## Methods

### Study design

In line with the explorative nature of our research aim, we chose a qualitative study design and used Grounded Theory. Grounded Theory is described as a suitable research method for investigating phenomena of social processes and for developing a theoretical framework [65, 66]. The Grounded Theory approach was based on the form proposed by Corbin and Strauss [65]. This form claims to outline a linear and structured approach, in which rules rather than interpretations play a significant role. It intends to make the analysis understandable, comprehensible and verifiable [66]. This research method provides a structured framework for the examination of a phenomenon about which little is known and can lead to the development of theory based on data. Sampling, data collection and analysis are defined as an interrelated and iterative process [65, 66].

### Framework

The study is based on a systemic approach. This approach considers interaction among humans as an object of psychological analysis and is therefore essentially focused on interpersonal systems [67]. This systemic model can be largely applied to all human interactive systems, its holistic view looks at people within the context of their environment [67]. Literature states chronic illness affects many activities of daily life [62, 68–71]. It not only affects the sufferer but also the surrounding social and familial systems [62, 69, 70]. We assume that coping with CPTSD in the social network cannot adequately be understood without sensitively considering the specific kind of relationship between sufferers and their relatives [45, 62, 69]. Our primary perspective is a systemic one in which we see the person affected as part of a system, a member of a group of people who interact together, who act and react in response to each other. In this study, we also subscribe to a central position of caregiver research. The foremost conceptual model for caregiving assumes that the onset and development of chronic mental illness may be stressful for both the patient and the caregiving person [35]. Caregiver research also examines the experience of supporting those affected, describes the nature of the relationships between different dimensions of caregiving [72–74], and addresses the caregiving relative as an expert [75]. Furthermore, we used the grounded theory coding paradigm to guide a linear and structured analysis of the data as well as aiming to explore the relationships in the data [66, 70]. That paradigm includes the dimensions: causal conditions, phenomenon, context, intervening conditions, action strategies, and consequences [65, 66].

### Setting

As part of a larger research project [64], this study was conducted at the psychiatric institution Integrierte Psychiatrie Winterthur - Zurcher Unterland (ipw). Ipw is a non-profit, community-based organization that provides psychiatric services in the city of Winterthur in the Canton of Zurich, Switzerland. The organization is responsible for approximately 440,000 inhabitants from the city and agglomeration of Winterthur. The clinic targets clients of all ages, from adolescents to the elderly. In 2017, a total of 3175 patients were hospitalized and about 800 staff were employed [76]. It provides a full continuum of clinic and community-based mental health services for individuals with various mental health issues. The current study was conducted at a specialized inpatient mental health ward for psycho-traumatology. The ward treats approximately 200 patients per year. It provides treatment for a diverse adult population from the German-speaking region of Switzerland. The ward has capacity for 17 patients.

### Participants

Over a ten-month period, CPTSD patients from a previous qualitative study (Stadtmann MP, Maercker A, Binder J, Schnepp W: Why do I have to suffer? Symptom management, views and experiences of persons with a CPTSD: A grounded theory approach, submitted) were chosen from a larger sample of another prior quantitative study (Stadtmann MP, Maercker A, Binder J, Schnepp W: ICD-11 complex posttraumatic stress disorder, characteristics and symptoms of adults in an inpatient psychiatric setting: A descriptive study, submitted). For each participant with CPTSD diagnosed according to the ICD-11 proposal, one relative was recruited. These relatives comprised the sample for this study. The Zurcher cantonal ethics review board approved the study. The current investigation comprised participants collected through theoretical sampling. Participants were required to meet the following criteria for inclusion: The patient has been diagnosed with CPTSD. The participants had to be adults, 18 years or older and with a good knowledge of German. Relatives were defined as persons who are self-defined relatives, who may or may not be bound by blood, law, friendship and who had declared commitment, shared deep personal connections with the participant and provided various forms of support in times of need [77, 78]. Exclusion criteria included providing support for a person affected with a main diagnosis other than CPTSD. Written informed consent was given prior to the interviews. The names contained in this manuscript are pseudonyms and have been changed to ensure the anonymity of study participants.

### Data collection and analyses

The first author, experienced in the treatment of and caring for patients with PTSD, was responsible for the qualitative data collection with semi-structured interviews. The first questions were developed based on the results of the prior qualitative study exploring the experience of persons affected by CPTSD relating to their symptom management (Stadtmann MP, Maercker A, Binder J, Schnepp W: Why do I have to suffer? Symptom management, views and experiences of persons with a CPTSD: A grounded theory approach, submitted). Similarly the domains of the symptom management model [60] were used to develop further questions. First the clinician’s internal code allowed the identification of patients, after which they were asked for an interview. Second, one relative was asked for an interview. Data were saturated after the fourteenth interview, which is when no new codes or themes were found. To secure data saturation, we conducted three further interviews. This extension adheres to current literature, which states approximately 15 participants are needed for data saturation to be achieved. The data saturation also adhered to the data collection and saturation of the first qualitative study exploring the experience of those affected. [79–81].

To create the largest possible contrasts between the interview partners, the participants were chosen using purposive sampling. The first three participants were identified based on the level of symptom burden of the patients (low, middle and high). Theoretical sampling was used as the study progressed and concepts emerged from the data. Thus, further participants were identified and chosen during the analyses, based on the questions arising during the process. For instance, relationships in which a single relative and the patient made up the unit were included in the study in contrast to cases with 3 or more relatives. Furthermore, we included blood and non-blood relatives as a contrast.

The interviews took place after the inpatient treatment of the person affected. The data collection took place between 1st March 2017 and 31st December 2017 at the psychiatric institution ipw in Winterthur, Switzerland. The semi-structured interviews lasted between 50 and 95 min; an additional 30 min after the interviews were available for questions and explanations. If any psychological crises had arisen due to the interviews, these could have been dealt with by a mental health nurse on the ward. The nursing team guarantees the shift organization on the ward 24 h a day.

The interviews were audio-recorded and subsequently transcribed verbatim. To comply with data protection, all names were anonymized. The semi-structured interviews were analysed based on the Grounded Theory proposed by Corbin and Strauss [65]. After using in a first step line-by-line coding with a group of Ph.D. students we were able to group the concepts into categories, in a second step the categories were reduced and clustered in the phase of axial coding. During the third coding level, the themes were finally selected and integrated into a final theory [31, 65, 66]. MAXQDA 12 software was used for this process. To ensure the quality of data analysis, the process itself and the results were not only analysed but also regularly discussed in a peer group of Ph.D. students led by an experienced qualitative researcher. Furthermore, the final model was member checked with all participants. The data analysis was undertaken in German, the language in which the interviews were conducted. The final report of the findings was written in English. To ensure reliable translation, triangulation with two bilingual persons (English and German) was performed.

## Results

**Fig. 1:**
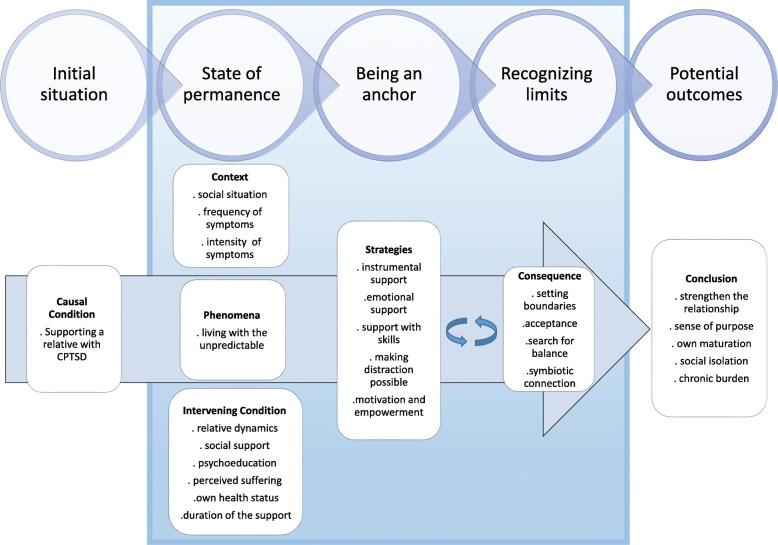
Process model for symptom management of relatives of patients with CPTSD

The data analysis resulted in a process with five categories or themes which we sorted into an explanatory framework that sequences the progression as well as the interactions and experiences of the relatives through their life whilst supporting a patient with symptoms of CPTSD. This framework (Fig. 1) is separated into five phases: 1.) initial situation, 2.) state of permanence, 3.) being an anchor, 4.) recognizing limits and 5.) potential outcomes. These sections each describe the characteristic categories and subcategories. We additionally provide participant quotes as examples (Table 1).

**Table 1:** Participants (pseudonym)

Name	Gender	Age	Occupation	Relation
Marc	m	45	gardener	husband
Sasuke	m	62	retail sales person	husband
Nicole	f	24	teacher	best friend
Amalia	f	32	no diploma or apprenticeship	girlfriend
Therese	f	23	arts student	daughter
May	f	28	law student	spouse
Jan	m	53	bank employee	boyfriend
Mikel	m	45	retail sales person	boyfriend
Ava	f	40	retail sales person	girlfriend
Hinata	f	43	office worker	best friend
Ami	f	32	registered nurse	sister
Rei	f	53	teacher	spouse
Mia	f	23	child-care worker	best friend
Adriana	f	35	hairdresser	best friend
Serenity	f	49	supervisor	sister
Kei	m	23	student natural science	best friend
Luna	f	28	office management	daughter

### Phase 1: Initial situation

This phase describes the **Causal Condition**. Being “supporting and caring for a relative with CPTSD” is given by the nature of the research aim. Most, but not all, relatives knew, even though not in detail, about the traumatic experiences of the affected persons. With time, they felt and thought something stressful had happened in their relative’s past, causing reactions they did not understand, for instance sudden shaking, sudden crying or having difficulties relaxing. If they did not know about the traumatic experience, they often tried to elaborate and make it a subject of discussion, while affected persons avoided the topic.

*“I know she has experienced many bad things. What exactly, I do not know, but I know she still suffers.” Jan, 53*

*“Yes, I know her diagnosis, I informed myself, so I could support her as good as I can. After all we are siblings.” Ami, 32*

### Phase 2: State of permanence

#### Main phenomena

In the literature, permanence is described as a state of lasting or remaining unchanged [82]. This phase is best explained by the main phenomenon of “living with the unpredictable”. For relatives it was most challenging to understand the condition of the sufferer. Often the question arose as to whether some reactions were mistakenly provoked through lack of empathy. Participants perceived the condition as unpredictable and the behaviour as incomprehensible. Relatives struggled to organize social activities as they were unsure whether the situation was tolerable for the affected person and whether they, themselves, could handle, for instance unreasonable behaviour, emotional outbursts or dissociative symptoms. Relatives worry whether the affected person might be in danger and being on standby was a permanent state. For example, while working, they often wondered whether the affected person was doing well or whether, for instance, they would receive an emergency call, or whether, in a stressful situation, the person affected would get lost and they would have to search for them. Further participants worried about when and whether the affected person would suffer from a relapse. The main phenomenon also included having concerns about the future.

*“And then she gets up and down. It’s like she is going high, high, high and she loses it and starts breathing. And she then like goes away. After half an hour she comes down. Sometimes it takes two or three hours.” Marc, 45.*

*“I was always aware, that was normal for me. I had to take care for my mum” Luna, 28*

#### Context

Context identifies factors such as “social situation”, “frequency of symptoms” and “intensity of symptoms”. Relating to the social financial situation, participants with a secure income described less ambiguity and stress regarding their future. Contrastingly, participants with neither secure income nor financial support experienced psychological distress. Thus, having financial issues was described as a major barrier. This situation was described as a context factor that made it difficult for relatives to cope with their daily life, to manage bills and to manage their administrative tasks.

Relatives living together with a sufferer of CPTSD more often had to handle demanding situations and reported experiencing a high frequency of symptoms, for instance, with sleep disturbances, outbursts of anger, inability to show affection, and a lack of energy to handle the household. Those symptoms had a high impact on the organization of daily routine. Individuals reported experiencing the need to manage symptoms nearly every day. While the frequency was high, the intensity varied. Most intense were visible symptoms, such as dissociation and self-injuries. These experiences were basically related to being present during the everyday life of those affected by CPTSD.

Contrastingly, relatives not living with the person with CPTSD reported having physical distance and living in another place as helpful in handling intense situations and symptoms. The context factor of living apart and distance had an influenced their perceived experience. However, those relatives also experienced the frequency and intensity as challenging and present when they spent time with their relative.

*“The worst is when I see new wounds. How can she… Even after years, I’m not sure how to react. Even though I should get used to it.” Adriana, 35*

*“Well it is not easy, she…, can’t work any longer. She can’t even do household chores. So, I have to take care financially that’s not easy.” Sasuke, 62*

#### Intervening conditions

The intervening conditions comprise perceived facilitators and barriers for symptom management in everyday life. The subcategory “relatives’ dynamics” comprised interactions between participants and the person with CPTSD. This subcategory can function as a facilitator as well as a barrier. The ability to learn to talk openly and respectfully with each other proved highly important. Those affected by CPTSD who shared some of their traumatic experience with their relatives helped the relatives to understand the cause of their condition and to better classify reactions. While in a first step relatives felt deeply shocked and unsure how to react, the information later provided orientation. Contrastingly, participants with no information about the disorder or the cause of the condition experienced disorientation and a lack of comprehension towards the person affected.

*“But, well, I feel I don’t understand him. He sometimes acts weird and aggressive and I feel it’s my fault, although I know it’s not. There must be something else.” Rei, 53*

Another subcategory was “social support”. Social support was described as formal social support. Here again, it was perceived as a facilitator as well as a barrier. If there were health care professionals or social workers involved, this was helpful and a major facilitator. While this support was described as a facilitator, relatives complained about the lack of involvement and information provided to them. When this involvement or information was absent, participants wished for formal social support not only for their affected relatives but also for themselves. This wish was most commonly based on lack of knowledge concerning the condition of their relatives, as described in “psycho-education”.

Major barriers were lack of information of the trajectory of the illness, what should be focused on, how to react best and the inability to make associations between the condition and some reactions. Another barrier was “perceived suffering”. Perception of visible suffering was a factor rendering it difficult to provide support for symptom management. Seeing a relative suffering inhibited possible support. This barrier was based on feelings of compassion, helplessness, sadness, lack of understanding and coping strategies. In general, relatives suspected persons with CPTSD of having more symptoms than they perceived or knew. If the relative’s “own health status” was impaired, for instance due to physical or mental illness, support for the relatives was also limited. Results reported in “duration of the support” were inconsistent. While some relatives experienced difficulties providing support at the beginning of the relationship but improved and became experienced with time, others reported feeling exhausted and burdened after years or decades of providing support.

*“She is like… a fairy. Not always comprehensible. She never was. But somehow we managed to master our life together.” Sasuke, 62*

### Phase 3: Being an anchor

#### Strategies

Being an anchor defines many different strategies for supporting the persons affected. The subcategory “instrumental support” describes how relatives took over tasks that had been neglected due to the illness trajectory. Duties included making dinner, doing the dishes, laundry, providing possibilities to rest, helping to structure the day and being a contact person for issues concerning children. Further tasks were taking care of financial issues, providing transportation, accompanying to doctors’ appointments and taking over administrative tasks, for instance with public authorities. Structures and predictable schedules helped to generate a sense of stability and security. Another subcategory described as “emotional support” refers to strategies such as minimizing stress at home or during daily routine, accepting the conditions patiently, not complaining at incomprehensible reactions, not pressurizing the affected person to explain every reaction, being there if that person wants to talk, and being a good listener. Further strategies were providing a feeling of being reliable and being a part of the affected person’s life. These strategies further imply expressing commitment to the relationship.

*“For me she is number one, and my friends are number 10. It is like it is.” Ami, 32*

*“He can relax at home, I mostly take care, so he can calm down.” Amalia, 32*

If the sufferer had knowledge of specific skills, they sometimes asked their relatives for assistance as described in the subcategory “support with skills”. Skills used were diverse. Some sufferers instructed their relatives to place a cold pack on their neck if they were dissociating, while others helped them to inhale an aroma therapy mixture. Often porcupine balls were used to deal with stressful situations. Prior to the assistance, relatives were informed where to find specific items, for example the cold pack, aroma therapy mixture, or massage tools, such as porcupine ball. Moreover, sometimes relatives also helped with taking medication, such as reminding the sufferer to take it or giving emergency medication during stressful situations. Relatives were required as an anchor to reality, for instance helping the sufferer to focus on their environment and to describe what they perceive around them. Likewise, relatives helped during a crisis to escort them out through a crowd.

*“I use ice. That’s something I grabbed hold of somewhere. We always have some ice in our freezer and I know when she is fading again I must use it. Not sure how. But it works.” Mikel, 45*

*“Well I have her medications and when she is fading away, I have to give her a pill, so she can come down.*” *Mikel, 45*

The subcategory “making distraction possible” was described as vital for relatives as well as for the people affected. The possibility of experiencing activities and situations not related to the condition strengthens personal ties and helps to ameliorate the sufferer’s symptoms. Another subcategory described as “motivation and empowerment” refers to efforts and attempts to keep the affected person encouraged and forward-looking. Described strategies were, for instance, motivating to go out for a short walk, going out for dinner despite feeling unwell, accompanying during shopping, or spending time with other friends. Furthermore, providing calming conversation during a crisis, pointing out strengths and resources and reminding of goals were described as motivational interventions.

*“I think it is important to be there. No one can be alone. Everyone needs someone right?” Nicole, 24.*

*“Sometimes it doesn’t need a lot. Just telling him he is doing great can help” Ava, 40*

### Phase 4: Recognizing limits

#### Consequence

During the process of support, relatives experienced a phase of possible consequences. The subcategory “setting boundaries” implies reactions such as learning to say no if they feel uncomfortable with a request, being able to speak with the affected person about their own needs and worries, redefining the time and resources spent with the sufferer and thus having more time for themselves. These strategies imply deciding the extent of support that can be provided. This support is, for example, redefining how much help is needed with a daily routine, for instance with cooking or washing the dishes.

“Well I know she is not well, but I also have to take care of myself and the children you know. So, I tell her sometimes I can’t, even though I might feel guilty afterwards” Marc, 45

Relatives realizing that they could not take on all burdens and recognizing that the person affected is also responsible for him- or herself is described in “acceptance”. This subcategory includes not only acceptance of the condition but also acceptance of one’s own limits and sometimes having mixed or negative feelings toward the sufferer. In this phase, participants described realizing the burden of caregiving and supporting.

This burden may lead to strategies in “search of balance”. This subcategory implies relatives’ strategies such as sharing responsibility and tasks with other relatives, for instance, with siblings or other friends and taking care of themselves with a support system (e.g. friends or people who share their hobbies), where the relatives could speak about their feelings and worries.

*“I love her, I really do. But I also love myself and I sometimes need distance between us” Serenity, 49.*

For some relatives setting boundaries was difficult, which led to a condition described as the evolvement of a “symbiotic connection”. The relationship was described as very close and intimate. Relatives felt they had to take responsibility for every aspect related to the well-being and the security of the person affected. Questions arose about whether the sufferer was sometimes in a bad condition because the relative had failed to support them adequately. If the relative failed to provide the desired support, feelings of guilt, shame, helplessness and emptiness arose for letting the sufferer down. The relative’s own needs, feelings, and necessities were in background. On the positive side, through providing support, feelings of satisfaction, a sense of value but also feelings of dependence were described.

*“She had no one else. I was there for my mum, always… and often I had to listen to stuff I didn’t really wanted to.” Therese, 23*

### Phase 5: Potential outcomes

#### Conclusion

Potential results whilst caring and providing support for a person with CPTSD are described as follows: being capable of mastering difficult experiences together as well as experiencing situations unrelated to the condition improved the quality of the relationship. Furthermore, sharing memories and experiencing mutual trust helped to nurture the relationship as described in the subcategory “strengthening the relationship”. Positive results arose not only for the relationship but also for individuals providing support. Helping a suffering relative also generated a “sense of purpose”. Thus, seeing the support as **meaningful work,** the desire to prevent negative effects for the person and feelings of being able to ameliorate symptoms were described as important for the relatives. Moreover, a sense of purpose includes meaningfulness in being there for a person in need, duty and obligation towards the sufferer and sometimes pride in being able to help and master various demanding situations together. The subcategory “own maturation” describes personal fulfilment, growth, and the mastery of new skills through the support, for instance, being able to handle a household, cooking, buying groceries and being a conversational partner for a person with CPTSD at an early age.

*“Well I guess I can’t compare our relationship with others. It is strong and trustful, after all we went through a lot of struggles.” Mia, 23*

On the other hand, negative results were also reported, for instance, “social isolation”. In this subcategory relatives felt secluded from their friends and social life. Supporting the person in need claimed not only a huge amount of time but also energy and personal resources. Difficulties in sharing experiences of tough situations when supporting a person with CPTSD were affected by either negative stereotypes, fear of negative stereotypes or by stigma towards those with a mental health condition. These feelings lead to the situation of having to deal, mostly alone, with rising emotions and distress.

*“It takes time being there, so I have less time for my other friends. I feel somehow alone.” Kei, 23*

Prolonged support with a lack of coping strategies, difficulties in balancing one’s own needs with the need to support and in pacing oneself led to a state described in “chronic burden”. Hereby relatives described the resulting condition as feeling exhausted, frustrated, sometimes angry with the sufferer, struggling with a bad mood as well as feeling emotionally empty and alone with the situation.

*“I really feel alone. But what should I do? I can’t leave him and at the same time it makes me really furious.” Rei, 53*

## Discussion

Emerging evidence suggest CPTSD to be a chronic condition in mental health setting [45, 83, 84]. Various chronic mental health conditions, such as schizophrenia or major depression have been described as sever and impairing [85, 86]. One of the most common characteristics of chronic mental illness is that psychiatric symptoms often not only reoccur and persist but can persist for the entire life of those affected [86–88]. Therefore, providing support and understanding symptom management process could be pivotal in improving the quality of life of those sufferers [89]. Literature describes relatives as an important and supportive part in the lives of patients with a chronic condition ([29, 71, 90]; Stadtmann MP, Maercker A, Binder J, Schnepp W: Why do I have to suffer? Symptom management, views and experiences of persons with a CPTSD: A grounded theory approach, submitted). We assume this interpersonal support might be of special importance in a condition such as CPTSD. Having a potential recovery effect on the loss of trust and possible lack of interpersonal skills based on prolonged and repeated experience of interpersonal trauma factors such as abuse in sexual, physical or emotional form, slavery or prisoners of war [5, 7, 45, 84]. We provide first results of experiences of relatives supporting the symptom management of persons suffering from CPTSD. Using the data collected through semi-structured interviews, we developed a conceptual model (Fig. 1) that identifies five major interacting but not linear phases experienced by supporting individuals: initial situation, state of permanence, being an anchor, recognizing limits, and potential outcomes. The results of our study confirm some of the results from studies with relatives of PTSD sufferers, especially results related to relatives’ difficulties with and burden of support [54, 58, 59, 91–94].

In our study, one difficulty is described as the main phenomenon of being under the constant state of living with the unpredictable condition. This result adds new findings specific to the context of CPTSD. The participants in this research criticized the lack of information, lack of involvement in therapeutic processes and the lack of formal social support. Hence, providing more professional medical knowledge and psycho-educational interventions might lead to clarification and an improved understanding of the condition.

The inability to understand the condition of the person affected is in line with several proposals to develop specialized family support and educational services [48, 95–98] that aim at adequate support such as cognitive–behavioural therapy, psycho-education, and problem-solving skills. These services would deliver more information and alleviate the burden associated with providing support. The absence of such support, however, may lead to negative effects for relatives, who jeopardize not only their capacity to provide support for symptom management but also their own health and well-being. We hypothesize, therefore, that relatives need ongoing access to information, guidance, and support to fulfil their role in symptom management effectively. Further, through guidance and tailored support they might be able to minimize potential risk to their own well-being.

Whereas our sample reflects heterogenic relationships providing a broad spectrum of experiences, views and strategies supporting affected persons, it raises the question of whether different social relationships experience different views, needs and strategies. For instance, are children of a parent with CPTSD in need of the same support and guidance as the spouse of a sufferer. Evidence from Mital and colleagues suggests there might be differences [39]. They state that there could be a difference in gender and in the way the support was provided to sufferers. Hence, further research aiming specifically at children or spouses could bring new and more precise findings. Also, the same authors provide results indicating that there is a significant relationship between the duration of the disease and possible isolation of the caregiving relatives. Our results concerning the duration of support were inconsistent. While some relatives reported feelings of exhaustion, others felt empowered and able to provide support. We hypothesize that the subcategories described in the phase “recognizing limits” might have an influence on those results. Hence, further and more specific research investigating a possible correlation between strategies and adverse results, such as social isolation or chronic burden, is required.

While previous research has examined the relationship between PTSD symptom severity and caregiver burden, this is the first study to gain insight into the social process of relatives providing support in symptom management for sufferers of CPTSD. It aimed to understand their views, needs and experiences and possibly to build up a basis for family-oriented support. Not all the described influencing factors can be changed or controlled. For instance, no supportive intervention for the relatives can influence the severity of the illness. However, how those affected handle their symptoms greatly influence their relative’s experience. Our results suggest that being able to reduce self-injuries or a better management of dissociative symptoms may reduce perceived symptom intensity and hence distress in relatives. The literature supports the assumption that the severity of the symptoms has a great influence on the relative’s experience in providing support, perceived burden, and the perceived severity of an illness [54, 91]. It seems, to relieve relatives, it is essential to also support persons with CPTSD in their individual process of managing their symptoms. The positive effect of providing this support for the sufferer is in line with a previous study (Stadtmann MP, Maercker A, Binder J, Schnepp W: Why do I have to suffer? Symptom management, views and experiences of persons with a CPTSD: A grounded theory approach, submitted), which suggests that the understanding of people affected by CPTSD with regard to interactions and reactions based on their illness gave them a sense of security to manage their symptoms; those affected described relatives as a key factor during this process. Thus, symptom management seems not to be an individual issue; it is more a holistic and systemic one. Our results indicate an essential need to develop specific mental health and family support for relatives helping with the symptom management of persons with the diagnosis of CPTSD.

## Conclusion

This study focused on the unique views and experiences of relatives providing support for symptom management in CPTSD. Participants experienced the condition as unpredictable. Although they mastered different strategies through own exploration and in cooperation, there is a clear need for more education, advice and support for relatives helping sufferers of CPTSD. Over all, well-supported relatives can play a facilitative and key role in improving symptom management.

Not every relative was automatically burdened or overwhelmed by providing support. For those who were, community health services should consider providing family support and educational services, thus leading to more involvement of relatives in treatment. Moreover, the unpaid informal support provided by relatives makes a major contribution to the health and social service system, which would be very costly to replace with paid formal support.

### Limitations

This research also has some limitations that should be considered in evaluating the results. Questions were designed by the first author; it can be assumed he is not value-free and that he inadvertently influenced the results due to his own professional and personal beliefs. Moreover, these values may influence how the researchers conducted and reported their research. Different findings might have been generated elsewhere by different researchers.

Based on our qualitative study design, our sampling was selective and purposive; it focused on participants with different kinds of relationships and socio-demographic factors identified during the ongoing analyses. Further contextual influences were not considered. Hence, the results cannot be generalized. Further, we do not know whether our results apply to other cultures, other countries or other settings in the Swiss health care system. We recognize that the conceptual model presented does not represent the unique symptom experiences of relatives providing support for those with CPTSD. In addition, the design of our study project might have a great impact on the results. The sample in this study was recruited on quantitative results of a first study and in dependence of CPTSD affected inpatients investigated in a second study. This structed not only could have provide holistic and supplementary results but could have created hidden bias.

We do not claim causal inferences based on our data and framework. As previously stated, further quantitative research testing our results is needed to determine possible cause and effects or correlations. Research is also needed to expand the conceptual model and to discover attributes specific to each phase. Despite these limitations, our study contributes primary results regarding the views and life experiences of relatives supporting the symptom management of persons affected and contributes to the expanding literature related to CPTSD.

## Abbreviations

CPTSD: Complex posttraumatic stress disorder
DGPPN: German Association for Psychiatry, Psychotherapy and Psychosomatics
DRKS: German Clinical Trial Register
Fig: Figure
ICD-11: International Classification of Disease, 11th version
ITQ: International Trauma Questionnaire
PTSD: Posttraumatic stress disorder
WHO: World Health Organization
